# Recent advances in precision oncology research

**DOI:** 10.1038/s41698-018-0055-0

**Published:** 2018-04-16

**Authors:** Ann M. Bode, Zigang Dong

**Affiliations:** The Hormel Institute, University of Minnesota, 801 16th Ave NE, Austin, MN 55912 USA

## Introduction

Precision oncology has evolved into focusing on matching the most accurate and effective treatments based not only on the genetic profile of the patient and his/her cancer, but also on other unique characteristics that distinguish one patient from another. Each patient has a unique genome, proteome, epigenome, microbiome, lifestyle, diet, and other characteristics that all interact to influence oncogenesis, disease progression, effective treatment options, drug tolerance, remission, and relapse. Cancer comprises several hundred heterogeneous diseases, meaning that differences exist not only between cancer cells from various patients, but also between cancer cells within a single patient. Cancer is constantly developing characteristics to evade death and this is why no single drug has been effective in “curing” cancer. Precision oncology now involves using a combination of the unique characteristics of each patient to direct immunotherapy and targeted therapies.^1^ Many advances in research over the past year reflect these ideologies of precision oncology. Here we describe a few recent policy and research advances in the field of precision oncology.

Most recently, the focus has shifted from treating cancer based on type and histology to treating the specific cancer mutation. FDA policies have paved the way for the adoption of “big data” genetic profiling techniques to guide patient therapies, such as those described by the NCI-MATCH (Molecular Analysis for Therapy Choice) or the NCI-MPACT (Molecular Profiling-based Assignment of Cancer Therapy) initiatives. In June 2017, the FDA granted regular approvals for using a combination of dabrafenib (i.e., a BRAF inhibitor**)** and trametinib (i.e., a MEK inhibitor) to treat patients if they had metastatic non-small cell lung cancer (NSCLC) with the BRAF V600E mutation. This regimen was first approved by the FDA in 2014 to treat melanomas in patients with BRAF mutations, and in 2017, researchers reported that the combination of dabrafenib and trametinib could also help patients with later stage melanoma by lowering the risk for recurrence after surgery.^2^ The hope is that the genetic profile of tumors can match patients with the BRAF V600E mutation treatment regimens including this combination or another BRAF inhibitor. Broader applicability remains to be seen but seems promising. However, results of next-generation sequencing have shown that most patients do not have “actionable” mutations like the BRAF V600E. In other words, we do not yet have a drug that can target every specific mutation identified. Also cancers can have more than one “driver” mutation. However, combination therapies seem to hold promise in overcoming these issues.

A major challenge in drug development has been the minimum 10-year time frame traditionally required to move a drug from Phase I through Phase II and III trials and eventually into FDA approval for use in the clinic. This time frame is no longer acceptable, and the FDA has developed the “Breakthrough Therapy Designation” (BTTD) and accelerated approval of protocols to speed up the process. The BTTD was first approved in 2012, and this strategy refers to a drug that can be used alone or in combination to treat a life threatening condition or disease. The drug must have preliminary clinical evidence indicating that its use could demonstrate substantial improvement over existing therapies on one or more clinically relevant endpoints. Drugs that are designated as breakthrough therapy are granted expedited review and development. Even though a therapeutic receives breakthrough designation, the drug must still be further assessed in additional trials to confirm its clinical benefit. If the additional trial confirms the initial result, then the FDA will grant regular approval. The FDA-accelerated approval program is one of four evidence-based strategies used to speed-up the evaluation of therapeutics to treat life-threatening diseases such as cancer. The accelerated approval is built around the assessment of the effect of a therapeutic drug at an earlier stage than usual by using a surrogate endpoint (i.e., a marker believed to predict clinical benefit, such as tumor shrinkage that is associated with longer overall survival or improved quality of life).

In spite of these challenges, 2017 was a remarkable year in the field of precision oncology and therapeutic advances. Notably, the European Society for Medical Oncology (ESMO) published a Precision Medicine Glossary in the Annals of Oncology.^3^ This publication is extremely important for establishing standardized communication regarding precision medicine between oncologists, researchers, and patients.

## Highlighted breakthrough designations

The aberrant activation of protein kinases is frequently a driver or a direct result of cancer.^4^ We know that many kinase-signaling pathways drive various hallmarks of tumor development such as survival, metabolism, motility, proliferation, angiogenesis, and evasion of immune surveillance.^5,6^ Thus, protein kinases are believed to be important targets for drug development, and in 2017, several new drugs and immunotherapies targeting protein kinases were granted either BTTD, accelerated or regular approval. One example, lorlatinib, an inhibitor of the ALK/ROS1 (c-ros oncogene 1) tyrosine kinase, was granted designation to treat patients with anaplastic lymphoma kinase (*ALK*)-positive metastatic NSCLC previously treated with one or more ALK inhibitors. Another example, osimertinib (Tagrisso), a third-generation epidermal growth factor receptor (EGFR) tyrosine kinase inhibitor, was granted designation for the first-line treatment of patients with metastatic *EGFR* mutation-positive NSCLC. Breakthrough therapies are commonly tested on patients with NSCLC because of poor overall survival rate. Many anticancer drugs act by inducing apoptosis or cancer cell death, and specifically targeting proteins that prohibit apoptosis is a key strategy. Venetoclax acts by blocking the anti-apoptotic B-cell lymphoma-2 protein (Bcl-2) leading to apoptosis of leukemia cells. This small molecule oral drug was granted designation in combination with low dose cytarabine (a chemotherapy compound that acts as an anti-metabolite) to treat older patients with previously untreated acute myeloid leukemia (AML) who are ineligible for intensive chemotherapy. However, blocking anti-apoptotic proteins can lead to a variety of adverse side effects, including low white blood cell and platelet counts, due to low or no specificity for which cells are affected by these drugs.

Antibody-drug conjugates (ADCs) have been developed as highly-potent, targeted therapies against various cancers. The intention of these drugs is to spare normal cells while specifically targeting and killing cancer cells. These complex molecules are comprised of a cytotoxic anticancer drug bound to an antibody that specifically and ideally targets a protein tumor marker only found on cancer cells. The antibody attaches itself to the target protein on the cancer cell surface, which causes a cellular response in which the antibody and the drug are absorbed into the cancer cell where the drug is released to kill the cell. At least three ADCs were granted designation: brentuximab vedotin, sacituzumab govitecan, and DS-8201. Brentuximab vedotin selectively targets CD30, which is highly expressed in Hodgkin lymphoma. This ADC is now used in combination with chemotherapy as a front-line treatment of patients with advanced cHL. The ADC sacituzumab govitecan (IMMU-132) targets trophoblast cell-surface antigen (Trop-2) and selectively delivers high doses of SN-38, the active metabolite of the topoisomerase 1 inhibitor, irinotecan. SN-38 is at least 1000 times more active than the parent irinotecan and causes DNA damage and cell death. Heavily pretreated patients with metastatic triple-negative breast cancer showed good response rates. The third ADC to receive designation was DS-8201 for the treatment of patients with HER2-positive, locally advanced or metastatic breast cancer who have received trastuzumab (Herceptin) and pertuzumab (Perjeta) treatment and have disease progression after therapy with ado-trastuzumab emtansine. The goal of ADC therapies is to increase specificity in order to decrease off-target effects, which will ultimately reduce the side effects that often accompany cancer therapeutics. While Breakthrough Designations will accelerate the production of these targeted drugs, the design of effective and safe molecules can be problematic.^7^

## New immunotherapies

Based on advances in genomic profiling, the fields of immuno-oncology and precision oncology are rapidly moving closer together.^8^ Immunotherapy was named in the ASCO’s Annual Report on the Progress Against Cancer as “Society’s Advance of the Year”. Researchers have been trying to harness the immune system for years in the fight against cancer with little success until recently with the discovery of immune checkpoint inhibitors and the development of CAR T-cell therapy. In August 2017, the FDA approved the first adoptive cell immunotherapy (i.e., CAR T-cell therapy) and the first gene therapy for cancer, tisagenlecleucel. Cancer cells have developed defenses to avoid immune cell detection and elimination. The recent immunotherapeutic approaches utilizing immune checkpoint inhibitors have successfully reversed the concealment of cancer cells from the immune system.^9^ Chimeric antigen receptor (CAR) T-cell therapy is a type of adoptive cell immunotherapy in which patient’s own immune cells are genetically reprogrammed to detect and kill cancer cells throughout the body. Tisagenlecleucel (Kymriah) is a genetically modified autologous T-cell immunotherapy, or CAR T-cell therapy, directed against cluster of differentiation 19 (CD19) antigens for B-cell acute lymphoblastic leukemia (ALL). CD19 is located on the surface of B linage cells, and when it binds to the antigen receptor on B-lymphocytes, it reduces the level for antigen receptor-dependent activation. In this treatment, T-cells are collected from an individual patient and genetically engineered to express a CAR that specifically targets CD19 and these cells are infused back into the patient. The CD19 CAR T-cells guide the T-cells to identify and kill leukemia cells expressing CD19 on their surface. The treatment is approved for patients up to 25 years old with B-cell precursor ALL that is refractory or in second or later relapse. The treatment has led to outstanding results in some young patients with ALL and in adults with lymphoma and multiple myeloma.^10^

On the downside, CAR T-cell therapy can induce an adverse side effect of cytokine release syndrome (CRS), which is a huge overproduction of inflammatory molecules. These molecules can result in high fever, lowered blood pressure, and respiratory problems, which can be reduced by treatment with tocilizumab, an immunosuppressive drug. Overall, CAR T-cell therapy is a combined immunotherapy and gene therapy, but, unlike traditional chemotherapy or other cancer treatments, CAR T-cell therapy is administered only once because CAR T-cells continue to multiply in the patient’s body thereby having the potential to consistently exert their anticancer effects indefinitely. This approach is being explored in other types of cancer, and in October 2017, the FDA approved another CAR T-cell therapy, axicabtagene ciloleucel (Yescarta), to treat adults with relapsed or refractory large B-cell lymphomas after two or more lines of systemic therapy. Another type of CAR T-cell therapy targets B-cell maturation antigen in multiple myeloma and was shown to be very promising in an early clinical trial.^11^

In 2017, the FDA also approved several new immunotherapies directed against programmed death-ligand-1 (PD-L1) or the programmed cell death protein 1 (PD-1). In contrast to CAR T-cell therapy, immune checkpoint inhibitor therapy targets an endogenous protein or receptor. PD-1 is a cell surface receptor that is a member of the immunoglobulin superfamily and is expressed on T cells and pro-B cells.^12^ PD-1 binds two ligands, PD-L1 and PD-L2 and several lines of evidence suggest that PD-1 and its ligands attenuate immune responses, which can prevent the immune system from killing cancer cells.^13^ PD-1 inhibitors and PD-L1 inhibitors are being developed as frontline treatment against several types of cancer,^14^ including advanced melanoma, NSCLC, renal cell carcinoma, bladder cancer, and Hodgkin lymphoma.^15^ They act by disrupting the association of PD-1 with PD-L1 and have been tested in more than 500 clinical trials involving more than 20,000 patients as of 2017.^13^

This past year, several PD-1 associated immunotherapies have been approved by the FDA, including avelumab, durvalumab, and nivolumab. Avelumab (Bavencio) is a PD-L1 blocking monoclonal antibody for treatment of metastatic Merkel cell carcinoma in patients 12 years or older. Durvalumab (Imfinzi) was granted accelerated approval for treatment of locally advanced or metastatic urothelial carcinoma in patients whose disease progressed following platinum-containing chemotherapy. Durvalumab is also a monoclonal antibody that blocks the interaction of PD-L1 with PD-1 and CD80. In December, the FDA approved nivolumab (Obdivo), a PD-1 monoclonal antibody to be used for adjuvant treatment of patients with melanoma metastasized to lymph nodes or in patients with metastatic melanoma who have undergone complete resection. Nivolumab was initially approved for treating patients with unresectable or metastatic melanoma. The Ventana PD-L1 Assay was approved for use as a diagnostic to assess the levels of PD-L1 in urothelial carcinoma paraffin-embedded tissues samples. Further development of diagnostic tools to identify candidate patients for PD-1 therapies will continue to broaden their use and development.

To date, pembrolizumab (Keytruda) appears to be the most versatile and widely tested PD-1 inhibitor. It was approved to treat classical Hodgkin lymphoma (cHL) in adult and pediatric patients with refractory cHL or who have relapsed after three or more previous therapeutic approaches. In May 2017, it was approved as first-line combination therapy with pemetrexed and carboplatin for patients with metastatic nonsquamous NSCLC, independent of PD-L1 expression and was also approved for patients with locally advanced or metastatic urothelial carcinoma that has progressed following platinum-containing chemotherapy. In the same month, pembrolizumab was first cancer treatment approved for any unresectable or metastatic tumor that expresses either microsatellite instability-high (MSI-H) or mismatch repair deficient (dMMR) biomarker. This is the first application of this therapy that was not prescribed based on tumor type, making it potentially applicable to a wide range of tumors. The FDA granted accelerated approval of pembrolizumab for patients with recurrent locally advanced or metastatic, gastric or gastroesophageal junction adenocarcinoma that express PD-L1. These approvals are a reflection of the plethora of groundbreaking changes in cancer treatment and the willingness of the FDA to assure that patients have faster access to these lifesaving treatments. These new emerging forms of cancer therapy promise to transform precision oncology. At the same time, safety and off-target toxicity, as well as development of resistance, are major concerns that must be considered. Important questions to be addressed are why some patients respond and others do not and why some patients respond initially to treatment, but eventually develop resistance. Notably, PD-1 or PD-L1 expression may or may not be associated with the response of all patients. Furthermore, whether these therapies can effectively target solid tumors is yet to be determined. One of the greatest challenges is identifying distinct proteins on cancer cells that can be specifically targeted without harming normal cells.

## Other highlighted FDA approvals

### Cyclin, kinase, and enzyme inhibitors

Use of protein kinase inhibitors to target cancer driver genes is a very effective tool for precision oncology.^8^ A flurry of activity has centered on the cyclin-dependent kinases, CDK4 and CDK6 (CDK4/6), which are commonly deregulated and over-activated in many types of cancer cells. A great deal of interest has focused on developing CDK4/6 inhibitors to attenuate growth-signaling pathways and restore control of cell cycle in cancer cells. CDK4/6 interact with cyclin D, which is synthesized at the start of G1 and these cyclin/CDK complexes drive cell cycle progression from G1 to S phase. Inhibitors block these kinases to stop the cells’ transition from G1 to S phase. In 2017, the FDA approved ribociclib (Kisqali) in combination with an aromatase inhibitor for treatment of postmenopausal women with hormone receptor-positive, HER2-negative advanced or metastatic breast cancer. Subsequently, abemaciclib (Verzenio) was approved for use in combination with fulvestrant, a selective estrogen receptor degrader (SERD), in similar patients but whose cancer had progressed after prior treatment. CDK4/6 have been extensively tested against breast cancer, but additional studies are underway in patients with liposarcoma, mantle cell lymphoma, NSCLC, glioblastoma (GBM), pancreatic adenocarcinoma, urothelial cancer, head and neck squamous cell carcinoma, metastatic castrate-resistant prostate cancer, ovarian cancer, and endometrial cancer.

Other notable approvals include several kinase inhibitors (Table 1). Notably the combination of dabrafenib and trametinib are the first FDA approvals specifically for treatment of patients with BRAF V600E mutation-positive metastatic NSCLC, targeting the mutation rather than the specific cancer. Besides kinases, many other types of proteins catalyze specific biochemical reactions in cells that are extremely important in cellular metabolism and other processes. These enzymes are normally tightly regulated transcriptionally and translationally and also at the activity level. At least three enzyme inhibitors were approved in 2017 for various indications (Table 1). Additionally, at least one antibody drug conjugate, inotuzumab ozogamicin (Besponsa), was approved to treat adults with relapsed or refractory B-cell precursor acute lymphoblastic leukemia. This drug targets CD22, a member of the SIGLEC (Sialic acid-binding immunoglobulin-type lectins) family of lectins found on the surface of B-cells, which inhibits B-cell receptor function.

**Table 1:** Additional kinase and specific enzyme inhibitors with FDA approvals

Drug name	Trade name	Mechanism of action	Application
*Kinase inhibitors*			
Neratinib	Nerlynx	Tyrosine kinase inhibitor	Adjuvant treatment early stage HER2 + breast cancer
Combination dabrafenib and trametinib	Tafinlar and Mekinist	BRAF inhibitor MEK inhibitor	Metastatic NSCLC with BRAF V600E mutation
Ibrutinib	Imbruvica	Burton’s tyrosine kinase	Marginal zone lymphoma
Acalabrutinib	Calquence	Burton’s tyrosine kinase	Mantle cell lymphoma in patients that have received at least one therapy
Midostaurin	Rydapt	CD135 (FLT3), c-kit, platelet-derived growth factor receptor, Src, and vascular endothelial growth factor receptor (VEGFR)	FMS-like tyrosine kinase 3 (FLT3) receptor mutation positive acute myeloid leukemia
Brigatinib	Alunbrig	ALK, EGFR wildype and mutant (T790M) proteins	Metastatic anaplastic lymphoma kinase (ALK)-positive NSCLC who have progressed on or are intolerant to crizotinib
Copanlisib	Aliqopa	Phosphatidylinositol 3-kinase (mainly PI3-Kα and δ isoforms	Relapsed follicular lymphoma
Rogorafenib	Stivarga	VEGFR2 and angiopoietin-1 receptor tyrosine kinase	Hepatocellular carcinoma who have been previously treated with sorafenib
*Other enzyme inhibitors*			
Teltristat ethyl	Xermelo	Tryptophase hydrolase (targets overproduction of serotonin inside mNET cells	Carcinoid syndrome diarrhea
Combination liposomal drug of daunorubicin and cytarabine	Vyxeos	Anthracycline topoisomerase and nucleoside metabolic inhibitor	Therapy-related acute myeloid leukemia (t-AML) and AML with myelodysplasia-related changes (AML-MRC)
Enasidenib	IDHIF	Isocitrate dehydrogenase inhibitor	Relapsed or refractory acute myeloid leukemia with IDH2 mutation

### Poly ADP-ribose polymerase (PARP) inhibitors

PARP inhibitors are one of the most promising new classes of oncology drugs that target the DNA damage response and DNA repair. The PARP enzyme is important in the repair of single-stand DNA breaks and inhibitors of PARP prevent DNA repair, leading to cell death. These inhibitors are especially effective against cancer cells exhibiting gene mutations that are critical in the process of homologous recombination, a process that is crucial for repairing double-stranded DNA breaks. For example, the presence of the *BRCA* mutation and treatment with a PARP inhibitor, which blocks single strand DNA break repair, will lead to cell death. Three PARP inhibitors have been approved by the FDA and include olaparib, rucaparib, and niraparib. Olaparib and rucaparib are used in patients with *BRCA-*mutated ovarian cancer who have received prior chemotherapy. Niraparib is used as a maintenance therapy for patients with platinum-sensitive recurrent ovarian cancer who are in response to platinum-based chemotherapy, regardless of *BRCA* or tumor homologous recombination status. At least one clinical trial has shown that olaparib was beneficial against breast cancer and more breast cancer trials are underway.^16^

## Major cancer initiatives

The year 2017 was noteworthy for the implementation of major new initiatives to fight cancer. The NCI-MATCH (Molecular Analysis for Therapy Choice) clinical trial is a precision medicine trial that is examining the idea of treating patients based on molecular profiles of the patient’s tumors regardless of the histology of the tumor. The trial has been accruing between 100 and 150 patients every week and is available at 1500 sites. The trial is open to adults with solid tumors (including rare tumors), lymphoma, and tumors that no longer respond to standard treatment.

The Beau Biden Cancer Moonshot was established to accelerate cancer research and make effective treatments available to more cancer patients in addition to improving prevention and detection methods. Major goals include encouraging more extensive cooperation and collaboration between researchers in academia, government and the private sector. The specific collaborative and cooperative goals include establishing a national network of patient biological and clinical data, increasing health disparity research, development of biomarkers, technology and preclinical research models and, importantly, methods of data sharing, analytics and predictive computational modeling. The Moonshot received $1.8 billion in promised funding over 7 years and $300 million was allocated for 2017. The establishment of the NCI Genomic Data Commons (GDC), aligned with the Moonshot, will provide all cancer researchers with a unified framework to sharing data, software, expertise, and communications.

Finally, the Department of Veteran Affairs, the Department of Defense, and the National Cancer Institute have formed a 3-agency collaboration referred to as the Apollo Network (Applied Proteogenomics OrganizantioaL Learning and Outcomes). This will enable oncologists to rapidly and accurately identify effective drugs to treat cancer based on a patient’s unique proteogenomic (i.e., combination of gene and protein expression) profile. The profile is developed by examining both a patient’s gene expression and the expression of proteins transcribed by these genes. The goal is to create the first U.S. system to routinely screen cancer patients for genomic and proteomic abnormalities to match targeted therapies to tumor type.

## New or expanding research in the field

### Nanotechnology

Two recent studies described two different methods for specifically targeting and killing cancer cells. The first was published in *Nature*^17^ by researchers from Durham University in the UK and showed that molecular machines using what the authors referred to as “nanomechanical action” could drill through cellular lipid bilayers to induce cell death. The molecular “motors” are adsorbed onto bilayers and activated by ultraviolet light to spin at 2 to 3 million rotations per second, opening cell membranes. Researchers showed that these tiny nanomachines could instigate the movement of chemical substances into and out of live cells or directly induce cell death. The motor was created in several sizes and could carry peptides designed to target and kill specific cancer cells. The nanomachines were tested in cell-based systems but they have yet to be tested in larger organisms.

The second study was a collaborative effort (Arizona State University scientists with researchers from the National Center for Nanoscience and Technology of the Chinese Academy of Sciences and from the QIMR Berghofer Medical Research Institute in Australia) that was published in *Nature Biotechnology*.^18^ In this study, investigators used DNA origami (i.e., nanoscale folding of DNA to create 2- or 3-D shapes on a nanoscale level) to create a DNA nanorobotic system to deliver thrombin to tumors. They showed that “nanorobots” (i.e., nanobots) could specifically target angiogenesis in cancer cells in mice to completely block blood supply. The nanorobots carried thrombin to tumor-related vessels resulting in intravascular thrombosis, which caused tumor necrosis and reduced tumor growth. Thrombin is a serine protease that causes the clotting of blood by converting fibrinogen into fibrin. Notably, the nanorobots effectively targeted tumor cells and not normal healthy cells. Blocking blood vessel growth has many advantages, including applicability to many types of solid tumors and decreased risk of developing resistance. This is the first report showing that nanometer-sized robots successfully delivered drugs to specific places to treat tumors in mammals. Each nanorobot was comprised of fragments of DNA arranged in sheets and four molecules of thrombin were attached to the surface. Thombin can reduce or prevent blood flow to the tumor by forming clots in vessels that feed the tumor. Each sheet was folded into a hollow tube shape to encase the thrombin protecting it from degradation and preventing its interaction with healthy cells. The nanobots were injected intravenously into mice where they traveled through the bloodstream to the tumor site. The nanobots were programmed to attach only to cancer cells by including a DNA aptamer (i.e., oligonucleotide or peptide molecule that binds to a specific target molecule) on their surface. In this case the DNA aptamer directly targeted nucleolin, which is specifically found on tumor-associated endothelial cells. Within 2 days the nanobots had successfully attached to tumor vessels, causing clots and inhibiting blood supply, resulting in tumor cell death. Notably, the nanobots only touched tumor cells and had no effect on normal cells. The nanobots have been tested in breast, melanoma, ovarian, and lung cancer mouse models and the hope is to move the technology into humans. Obviously, this is potentially a major breakthrough for cancer treatment if it can be effectively translated into humans.

### A novel way to kill cancer cells?

Many anticancer therapies induce cancer cell death through a process called apoptosis, which requires the activation of proteins called caspases. Apoptosis is usually initiated by disruption of the mitochondrial membrane potential, which results in mitochondrial outer membrane permeabilization (MOMP). The process of apoptosis does not always kill all cancer cells, which can lead to recurrence and sometimes results in increased cancer progression. A new and promising area of research involves a process referred to as caspase-independent cell death (CICD) in which cells die following MOMP even in the absence of caspase activity. This work was recently published in *Nature Cell Biology*.^19^ To study and compare apoptosis and CICD, investigators used a model system of combining the small molecule B-cell lymphoma 2 (Bcl-2) homology 3 (BH3) mimetic ABT-737 with or without the pan-caspase inhibitor Q-VD-OPh. The BH3 mimetic functions by binding to multiple members of the anti-apoptotic Bcl-2 protein family to inhibit their activity and restore apoptotic processes in cancer cells. Investigators observed that ABT-737 treatment caused cell death whether or not caspases were active. ABT-737 appeared to be dependent on MOMP because cell death was prevented by expression of anti-apoptotic MCL-1, which is not sensitive to ABT-737. Investigators determined that necroptosis, a programmed form of necrosis or inflammatory cell death, was likely engaged as a form of CICD. Furthermore, MOMP-stimulated necroptosis requires pro-inflammatory tumor necrosis factor (TNF) signaling, which is increased in CICD. Importantly, in the absence of active caspases, treatment with ABT-737 significantly increased activation of NF-κB, which was dependent on MOMP. Additional data demonstrated that MOMP could signal inhibitor of apoptosis (IAP) degradation and up-regulation of NF-κB-inducing kinase (NIK). Cells undergoing CICD promoted inflammatory activation of macrophages that was dependent on MOMP-dependent NF-κB activity. Studies in mouse models revealed that engaging CICD in tumors resulted in inflammatory and immune effects and, notably, almost complete tumor regression. Investigators concluded that the anti-cancer effects of immunogenic CICD require a healthy immune system and NF-κB activation.

### CRISPR/Cas9 clinical trials in the U.S.?

A posting for a CRISPR/Cas9 clinical trial is listed on the NIH directory of trials (Clinical Trials.gov), which indicates that these trials are still in the recruiting phase. The University of Pennsylvania had received permission from a federal panel to pursue the first trials in the U.S. using the CRISPR gene-editing system. This is planned as a Phase 1 trial looking to recruit 18 patients with multiple myeloma, melanoma, synovial sarcoma or myxoid/round cell liposarcoma. Scientists propose to use the CRISPR DNA-cutting technique to delete programmed death-1 (PD-1) receptor and remove and replace defective T-cell receptors (TCR) with a new receptor that is engineered to sound the alarm against specific cancers (NYCE T-cells). Blood will be collected from patients and T-cells will be isolated and edited with CRISPR before re-infusion back into the subjects. Basically, the study will convert normal human immune cells into cancer cell killers and University of Pennsylvania officials hope that the trial can start very soon in early 2018. Additional trials are planned in Europe at a later time. The first trial involving CRISPR was initiated in 2016 at the West China Hospital in Chengdu. In this trial, immune cells from a patient with lung cancer were used and only PD-1 was disabled before cells were returned to the patient. Primary outcomes include safety profile of a single infusion of engineered NYCE T-cells and evaluation of the feasibility for manufacturing NYCE T-cells. Secondary outcome measures will include percentage of patients achieving complete response, overall survival, duration of remission, progression-free survival and cause of death when appropriate.

### Progress against glioblastoma (GBM)

Two new strategies were developed that could potentially lengthen life for people with GBM. The first strategy involves a novel technology known as tumor-treating fields (TTFs). TTFs are low-intensity electrical fields believed to be able to put the brakes on cancer growth by blocking cell division. The electrical fields are administrated to the GBM through the skin from a device that patients wear on their head continuously at least 18 h a day. The FDA approved the device in 2015 based on preliminary findings from a clinical trial of TTFs. The device was approved for use in combination with temozolomide (i.e., alkylating agent) chemotherapy, after surgery, chemotherapy, or radiation, for patients with newly diagnosed GBM.^20^ In 2017, researchers reported longer follow-up findings from the same clinical trial. The median survival of patients who used the device was increased compared to those who received only chemotherapy the 5-year survival rate more than doubled from 5 to 13%. The second major advance against GBM was the discovery that adding temozolomide chemotherapy to short-course radiotherapy resulted in longer survival than radiotherapy alone in elderly patients with GBM.^21^

### Progress in big data handling

A proof-of-concept study used a beta version of Watson for Genomics technology to assist in the interpretation of whole-genome sequencing data from a sample of GBM from one patient.^22^ The goal was to analyze the sample with three different platforms and compare potentially actionable calls from each platform. The platforms included analysis of DNA by a commercial targeted panel, whole genome sequencing (WGS) and RNA sequencing (RNA-seq). Data obtained by WGS and RNA-seq were analyzed by oncologists and bioinformatics experts or by IBM Watson Genomic Analytics (WGA). The WGA is an automated system that prioritizes somatic variants and identifies drug candidates. Variant identification was best by WGS/RNA and, and notably, WGA completed the analysis in 10 min compared to 160 h of human analysis. Thus, combining WGA with manual analysis could effectively assist physicians in identifying potential therapies from WGS in less time for more patients.

### Liquid biopsies: Can they be useful?

Research has shown that tumor-derived changes in DNA can be detected as cell-free DNA (cfDNA) in plasma from cancer patients^23^ and are generally not found in normal subjects.^24^ Although challenging, circulating tumor cells or circulating tumor-derived cfDNA appear to have potential to be utilized as prognostic or predictive biomarkers in monitoring patient therapeutic response. Technology is being developed that will allow more sensitive and specific sequencing of cfDNA from plasma. This technique of sequencing cfDNA is referred to as a liquid biopsy and could have several advantages over tumor sequencing. Tumor biopsies are quite invasive and are not very repeatable in patients, whereas the liquid biopsy requires only a blood sample that can be repeated more than once. Liquid biopsies have the potential to efficiently monitor treatment response and might be used as an early-stage detection tool.^25^ However, knowledge regarding the physiology or biology of cfDNA, where it originates and how it moves into the circulation is sparse and obviously, more research is needed to answer these questions.

## Conclusion

### Where are we headed?

Precision oncology now encompasses precision immunotherapies, precision informatics, precisely targeted therapies, and specific cellular engineering all based on next-generation sequencing and genomic profiling. Emerging clinical cancer treatments are progressively and increasingly combining different and varied targeted therapies and pairing those therapies with traditional therapies like chemotherapy and radiation to address the many faces of cancer. Routine genomic profiling will become more affordable and many believe that it will improve diagnosis and prognosis for the cancer patient. The hope is that genetically-driven tumor dependencies and weaknesses will lead to effective precision combination therapies. However, one of the greatest challenges in genetic profiling is the interpretation of the results. Changes will be observed in a plethora of genes but which changes will be considered “actionable” or functionally relevant or biologically significant? In addition, many of the changes observed might be the result of therapeutic treatment that can result in the activation of off-target pathways to bypass the intended targeted pathway. How will resistance be addressed, which occurs almost universally with targeted cancer therapies? Resistance is a complex issue that can involve reactivation of the targeted pathway through additional genomic mutations either in the targeted gene or another, which can result in bypass of the intended target and tumor growth. This knowledge has led to multi-drug therapies to maintain efficacy. What are the variables that are most important for the successful implementation of precision oncology in the clinic? Clearly, we must identify proven, validated therapeutic targets and develop drugs that directly interact with those targets resulting in their deactivation and elimination. We must understand the mechanisms underlying resistance. Perhaps, techniques such as liquid biopsies could be key in understanding resistance based on cfDNA samples obtained from the tumor before treatment, throughout treatment, and after treatment.

Precision oncology clearly comprises more than just genomic profiling. These insidious multifaceted diseases are capable of continually adapting to and interacting with a harsh microenvironment, which we do not totally understand, to survive and even thrive. Anti-cancer drugs are being rapidly developed, tested and approved by the FDA in an expedited effort to get the drugs to as many patients as possible in the shortest and safest timeframe. Combinations of therapeutics to attack multiple cancer signaling pathways are rapidly being seen as more effective than a single drug treatment because of eventual acquired resistance. Immunotherapies have made a significant and positive impact on survival and quality of life in some patients. Even so, resistance is also an issue with these therapies. Federal financial support of new national anticancer initiatives, such as NIH-MATCH, Beau Biden Cancer Moonshot and APOLLO has increased dramatically. New technologies on the horizon, such as nanobots, CRISPR/Cas9 therapies, liquid biopsies, and advanced data computational analysis by systems like Watson, all hold great promise for progress in precision oncology. However, practically speaking, one institution or one agency cannot be solely responsible for every aspect of comprehending the complexities of cancer. The realization of effective and efficient precision oncology will not work if institutions do not talk with each other. Understanding these diseases and how to treat them precisely will absolutely require a tremendous collaborative and integrative effort between research and medical institutions worldwide.
